# Fibre Tip Sensors for Localised Temperature Sensing Based on Rare Earth-Doped Glass Coatings

**DOI:** 10.3390/s141121693

**Published:** 2014-11-17

**Authors:** Erik P. Schartner, Tanya M. Monro

**Affiliations:** ARC Centre of Excellence for Nanoscale BioPhotonics and Institute for Photonics and Advanced Sensing and School of Chemistry and Physics, The University of Adelaide, Adelaide 5005, Australia; E-Mail: tanya.monro@adelaide.edu.au

**Keywords:** rare earth, thermometry, point sensing, optical fibres, optical fibre sensing

## Abstract

We report the development of a point temperature sensor, based on monitoring upconversion emission from erbium:ytterbium-doped tellurite coatings on the tips of optical fibres. The dip coating technique allows multiple sensors to be fabricated simultaneously, while confining the temperature-sensitive region to a localised region on the end-face of the fibre. The strong response of the rare earth ions to changing temperature allows a resolution of 0.1–0.3 °C to be recorded over the biologically relevant range of temperatures from 23–39 °C.

## 1. Introduction

Optical fibre temperature sensors present an attractive alternative to traditional measurement techniques, as their small size and immunity to electrical interference enables their use in regions in which it would be difficult or impossible to obtain measurements with conventional methods. Multiple optical measurement methods have been demonstrated in recent years, with the majority of these centred on methods, such as Bragg/long period gratings or resonance-based techniques [1]. However, for applications requiring the temperature to be monitored with high resolution at a specific location, these methods are not ideal. Bragg gratings display a relatively low response to temperature, which limits their resolution [2]; long-period gratings require long fibre lengths to obtain good sensitivities, thus limiting their spatial resolution [3]; and whispering gallery mode (WGM) techniques are typically also sensitive to changes in the local refractive index [4]. While alternative methods for grating fabrication [5] can provide excellent sensitivity with good spatial localisation, their fabrication complexity makes it difficult to expand these to commercial applications where a cheap or disposable sensing element is required.

It has previously been shown that emission from rare earth ions, such as erbium [6–8], neodymium [9], praseodymium [10], europium [11] or holmium [9], doped within a suitable host medium depends on temperature. As the temperature of the medium changes, the populations in the two thermally-linked states change as ions undergo thermal excitation to the higher energy state [9]. By monitoring the upconversion emission from the ions, this process can be used as an indicator of the temperature of the host medium [12]. By using a ratiometric detection method, where the emission intensity in two specific bands is compared to each other, the temperature information can be extracted independent of the excitation power, greatly simplifying the measurement procedure compared to intensity-based techniques [9].

Co-doping of the glass with a sensitiser allows the upconversion efficiency to be dramatically increased compared to a glass doped with just the active ion, as resonant energy transfer can occur between particular energy levels [7]. In the case of erbium, co-doping with ytterbium increases the green upconversion efficiency due to the large absorption cross-section of Yb^3+^, greatly reducing the excitation power required to generate a given fluorescence signal [13,14]. This reduction in power is important for biological measurement applications, as it minimises the risk of cell death or damage, while still maintaining the autofluorescence-free advantage provided by upconversion-based techniques [15].

Previous methods using upconversion emission as a temperature indicator have typically been based on bulk glass samples, uniformly-doped optical fibres or doped microspheres [15,16]. While doped fibres have some advantages, primarily that they can be used to perform distributed measurements, they typically preclude high-resolution measurements being performed at the tip of the fibre, since a large background signal is present from the length of fibre not located at the desired sample location. Solutions, such as splicing lengths of doped fibre for excitation and collection, have been explored [9]; however, such approaches require extensive post-processing of each sample, greatly increasing the cost and time required to fabricate each probe.

To overcome this issue, we propose the use of rare earth-doped tellurite glass [14,17,18], into which the tips of silica fibres are briefly immersed to sensitise the fibre tips to temperature. This allows the physical size of the temperature-sensitive region to be minimised, thus greatly increasing the spatial precision with which the temperature can be measured. This fabrication method also significantly reduces the processing time required to fabricate each probe compared to alternatives, such as splicing or gluing methods. Using this technique, multiple probes can be fabricated simultaneously, with minimal preparation of the individual fibres required.

## 2. Experimental Section

### 2.1. Glass Preparation and Coating

**Figure 1.**
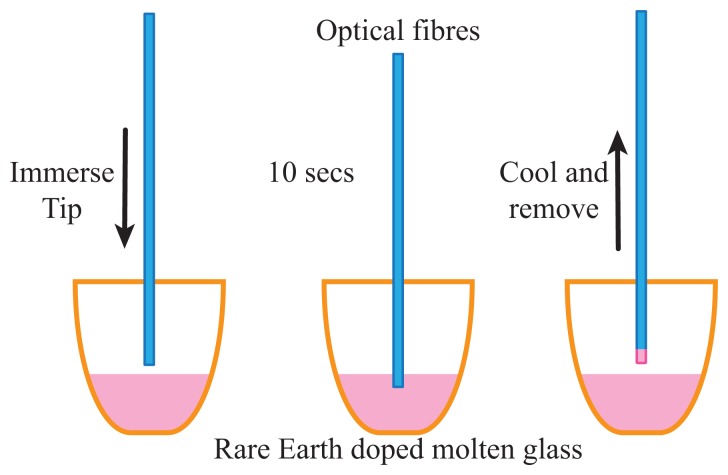
Fibre coating method, in which the fibre tip is briefly immersed within the molten glass to form a thin layer of active temperature-sensitive tellurite glass on the end face of the silica fibre.

Sensors were fabricated from commercially available Corning multi-mode fibre, with a core diameter of 62.5 μm and an outer diameter of 125 μm. For temperature sensing measurements, the glass host material chosen was sodium zinc tellurite (ZNT) glass [19], doped with 1 mol% erbium and 9 mol% ytterbium. This concentration was chosen as a compromise between maximising the intensity of the emitted upconversion signal and the expected decrease in the thermal sensitivity at higher doping concentrations [20]. The coating method used to fabricate the sensing region on the fibre tips is shown in Figure 1. Tellurite glass here was used for the fabrication of the tips, as the melt temperature used here (850 °C) is significantly below the softening point of the silica glass fibres (≈1600 °C), so no deformation of the fibres is observed.

The protective polymer layer was first removed from the region of the fibre to be coated using mechanical strippers and the fibre cleaved using a diamond cleaver. The tellurite glass was melted at 850 °C in an open air furnace and the crucible removed from the furnace for the dipping process. Fibre samples were mounted with their tips level and immersed briefly in the molten glass at a depth of approximately 5 mm using a mechanical stage to control the dipping process. The thickness of the coating can be altered simply by changing the melt temperature, as the viscosity of the molten glass has a large influence on the resultant coating thickness.

**Figure 2.**
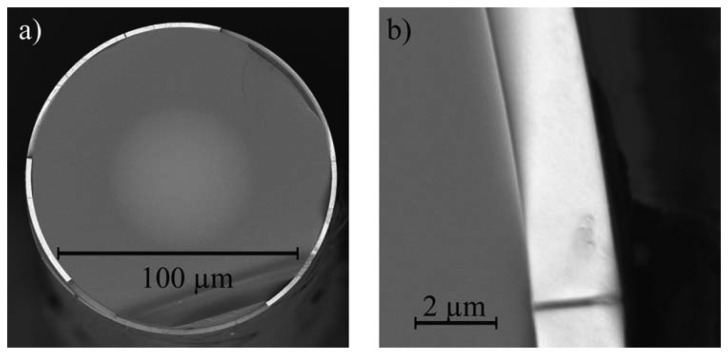
(**a**) SEM image of a Er Yb coated multi-mode fibre, cleaved to show the higher index tellurite layer surrounding the silica fibre. (**b**) High magnification image showing the outer coating layer thickness of approximately 2.2 μm.

In addition to coating the end face of the fibre, the tellurite glass coating is also applied to the sides; however, this region does not interact with the excitation light and, as such, has no influence on the detected signal. Figure 2 shows an example of a coated fibre, where the fibre has been cleaved a short distance from the end face to show the outer coating layer. As can be seen in Figure 2b, the thickness of the tellurite coating on the outer surface of the fibre is approximately 2.2 μm.

### 2.2. Temperature Measurements

**Figure 3.**
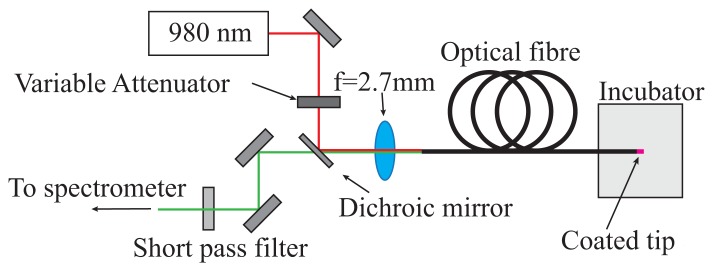
Experimental configuration for fibre optic temperature measurements.

Temperature measurements were performed using the optical setup shown in Figure 3. A 980-nm laser diode was used for excitation of the doped fibre tips, with power levels varied from 100 μW to 1 mW, depending on the observed fluorescence from the probe. The detector used was an Ocean Optics QE65 Pro detector, with the upconversion emission coupled to the spectrometer using a 400-μm diameter multi-mode fibre.

A resistance temperature detector (RTD, 100 Ω Class A) was co-located within 500 μm of the fibre within the incubator to allow calibration and testing to be performed. The incubator was then temperature cycled directly from room temperature to 39 °C and back to record the thermal response of the sensor probe. Due to the high thermal mass of the incubator, the heating rate is relatively slow, with the incubator taking approximately 55 min to reach maximum temperature. As such, the assumption that the temperature probe and RTD are in thermal equilibrium can be made, without requiring the temperature to be raised stepwise.

National Instruments LabVIEW software was used to simultaneously record both the reference temperature from the RTD and the upconversion emission spectra from the fibre probe. This program then integrated the fluorescence spectra over the desired range, allowing the comparison of the reference temperature and fluorescence ratio to be performed in real time.

## 3. Results and Discussion

**Figure 4.**
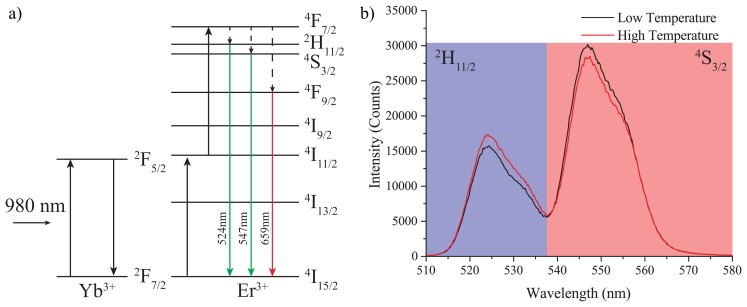
(**a**) Erbium ytterbium energy level diagram. (**b**) Example erbium ytterbium spectra for the two measured fluorescences. The two bands recorded to measure the fluorescence intensity are shaded in red and blue. Both low (23 °C, black) and high (39 °C, red) temperature emission spectra are shown, demonstrating the variation in the emission ratio of the two bands with changing temperature.

Fluorescence spectra obtained from the probe are shown in Figure 4b, with the integrated regions for the fluorescence ratio shaded in blue and red for the first and second fluorescence bands, respectively. The blue region corresponds to the ^2^H_11/2_ → ^4^I_15/2_ Er^3+^ transition, while the red corresponds to the ^4^S_3/2_ → ^4^I_15/2_ Er^3+^ transition, as shown in the energy level diagram in Figure 4a. A third strong red upconversion band (^4^F_9/2_ → ^4^I_15/2_) is also observed from the fibre probes, but is not used here in the measurement of the fluorescence ratio.

The fluorescence intensity ratio (FIR) here is then defined as the sum of the ^2^H_11/2_ → ^4^I_15/2_ band divided by the sum of the ^4^S_3/2_ → ^4^I_15/2_ band. This ratio has been shown in the literature to be well correlated with the temperature of the glass host [6,18].

**Figure 5.**
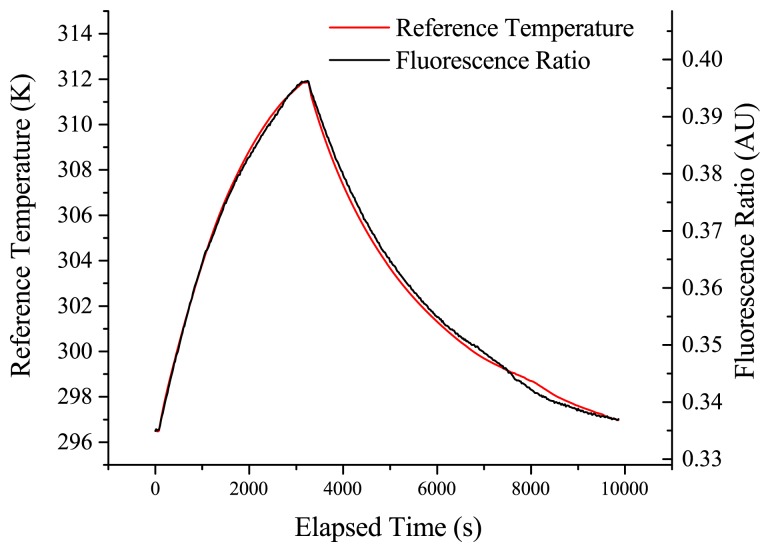
Erbium ytterbium-doped multi-mode fibre response with a co-located fibre probe (black) and reference resistance temperature detector (RTD) (red) inside an incubator. The fluorescence ratio is shown scaled to the maximum and minimum values recorded on the reference temperature.

The results shown in Figure 5 show a good correlation between the temperature and the fluorescence ratio of the erbium:ytterbium-doped sample over the duration of the measurement. The excitation power for this trial was 300 μW.

**Figure 6.**
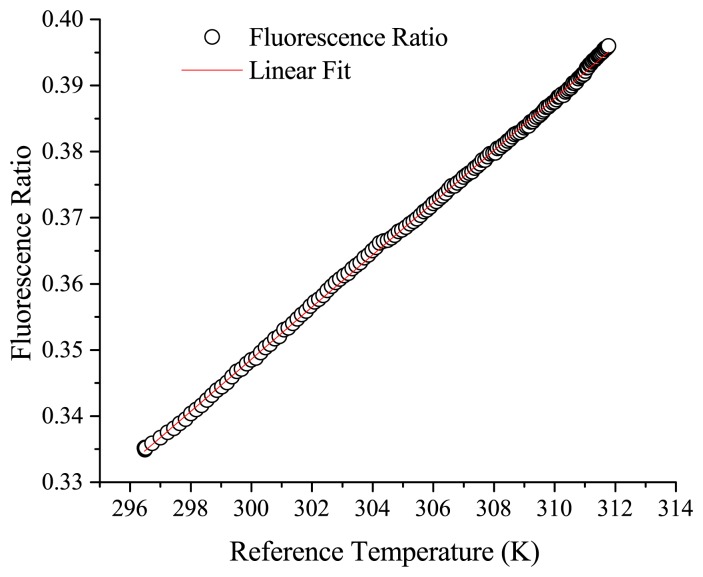
Fluorescence ratio *vs.* reference temperature for increasing temperature, R^2^ = 0.99942.

Slight separation between the observed fluorescence ratio and the true temperature is seen over the duration of the measurement, with a maximum deviation of approximately 0.2 °C observed in this trial between the fibre probe and reference RTD. This is most likely due to the drift of the alignment into the spectrometer due to fluctuations in the laboratory conditions. This drift could potentially be reduced by removing the free space optics and using a completely fibre-based setup for coupling the excitation light in and emission light out of the probe fibre. The probe responds well to repeat thermal cycling, again with some drift from the reference value observed over long experimental durations. A plot of the fluorescence ratio *versus* the recorded reference temperature for the 23 °C to 39 °C segment is shown in Figure 6.

This plot demonstrates that the measured change in the fluorescence ratio can be approximated as linear over the measured range, with a sensitivity (dFIR/dT) of 0.00394 K^−1^ and an R^2^ of 0.99942 over the biologically relevant range recorded here from 296 to 312 K. These results show that the short-term temperature resolution is <0.1 °C, while the accuracy is limited to approximately 0.1–0.3 °C by the long-term drift from the reference value.

## 4. Conclusions/Outlook

We have successfully demonstrated a new approach to localised temperature measurement based on a probe at the tip of an optical fibre. This device exhibits a temperature resolution of approximately 0.1–0.3 °C, which is comparable to other upconversion-based techniques previously demonstrated in the literature [15].

By utilising a relatively thin layer with a high dopant concentration, the required excitation power is minimised (300 μW), reducing the potential for damage to biological samples [15]. This implies that the excitation density of this sensor is approximately 98 mW/mm^2^, which compares well to literature values for power densities [21].

While the temperature range examined in this work primarily covers the biologically relevant region, this could, in principle, be extended to the thermal limits of the tellurite glass (T_g_ ∼ 300 °C [17]). If higher temperatures still were desired, then the glass choice could be altered to a soft glass, such as lead silicate glasses (F2 glass T_g_ = 434 °C [22]), which would increase the potential measurement range.

As the tip of a typical fibre is only 125 μm, with the temperature sensitive region localised to a thin area on the end face of the fibre, these probes should enable measurements to be performed in regions that are otherwise difficult to access, for example in *in vivo* or *in vitro* applications where the use of a conventional thermocouple is not possible. Probes with significantly reduced outer diameters (5–10 μm) have also been fabricated via tapering and coated with doped tellurite glass, demonstrating the potential for creating probes that can perform measurements in even smaller samples.

The NIR excitation also ensures that these measurements will not be affected by autofluorescence from the biological samples, which is potentially an issue when using visible excitation sources [15]. This is especially important for *in vivo* measurements, where autofluorescence from the surrounding tissue limits the potential sensitivity of standard fluorescence-based techniques.

Additionally, the detection method could be further simplified for a lower-cost implementation, as the two emission bands could be compared through the use of basic filters and photodiodes, rather than the high-cost spectrometer used for these measurements. This, combined with the eye-safe laser powers used in these experiments, should allow deployment of these sensors to biological or medical research labs that are not typically equipped to handle higher-power laser sources.

The method of fabrication demonstrated here allows multiple sensors to be fabricated simultaneously, without the requirements of individual post-processing that are typical for alternative methods of fabricating optical fibre temperature sensors. This work has demonstrated simultaneous fabrication of up to 10 probes in a single dipping trial, with the potential to further scale this depending on application requirements. These probes have the potential for employment for *in vivo* measurements, as the combination of the localised temperature-sensitive region with the minimal excitation power requirements and background-free upconversion emission will allow their use for real-world measurements.
